# Assembly of the Murine Leukemia Virus Is Directed towards Sites of Cell–Cell Contact

**DOI:** 10.1371/journal.pbio.1000163

**Published:** 2009-07-28

**Authors:** Jing Jin, Nathan M. Sherer, Gisela Heidecker, David Derse, Walther Mothes

**Affiliations:** 1 Section of Microbial Pathogenesis, Yale University School of Medicine, New Haven, Connecticut, United States of America; 2 HIV Drug Resistance Program, National Cancer Institute-Frederick, Frederick, Maryland, United States of America; Fred Hutchinson Cancer Research Center, United States of America

## Abstract

We have investigated the underlying mechanism by which direct cell–cell contact enhances the efficiency of cell-to-cell transmission of retroviruses. Applying 4D imaging to a model retrovirus, the murine leukemia virus, we directly monitor and quantify sequential assembly, release, and transmission events for individual viral particles as they happen in living cells. We demonstrate that de novo assembly is highly polarized towards zones of cell–cell contact. Viruses assembled approximately 10-fold more frequently at zones of cell contact with no change in assembly kinetics. Gag proteins were drawn to adhesive zones formed by viral Env glycoprotein and its cognate receptor to promote virus assembly at cell–cell contact. This process was dependent on the cytoplasmic tail of viral Env. Env lacking the cytoplasmic tail while still allowing for contact formation, failed to direct virus assembly towards contact sites. Our data describe a novel role for the viral Env glycoprotein in establishing cell–cell adhesion and polarization of assembly prior to becoming a fusion protein to allow virus entry into cells.

## Abstract 2

Applying 4D imaging, this study investigates the mechanism by which cell-cell contact enhances retrovirus spreading and demonstrates that viral budding is highly polarized towards sites of cell-cell contact.

## Author Summary

Retroviruses such as the human immunodeficiency virus are known to spread much more efficiently under conditions of direct cell–cell contact as compared to cell-free conditions. How cell–cell contact stimulates virus spreading is poorly understood. In this study, we apply four-dimensional imaging (3D space over time) of a model retrovirus to directly monitor and quantify the events of assembly, release, and transmission of individual viral particles in real time in living cells. Our work reveals that after contacts are established between virus-producing cells and uninfected target cells, the majority of virus particle assembly is initiated at sites of cell–cell contact. The ability of the virus to direct assembly of its particles towards sites of cell–cell contact is dependent on the presence of the cytoplasmic tail of the viral envelope glycoprotein. When this cytoplasmic tail was deleted, virus assembly at cell–cell contacts was no longer enhanced. This study contributes to an emerging model in which several steps of the viral life cycle are efficiently coordinated at sites of cell–cell contact, thereby promoting the spreading of viral infection to neighboring cells.

## Introduction

The ability of retroviruses to utilize and manipulate cell–cell contact for the purpose of efficient transmission contributes to the spread of infection and the progression to diseases such as leukemia and AIDS. In vitro, cell-to-cell transmission of the human immunodeficiency virus (HIV) is 100–10,000-fold more efficient under conditions of direct cell–cell contact as compared to cell-free virus [1]–[4]. The spread of the human T cell leukemia virus 1 (HTLV-1) depends on contacts between lymphocytes, and little cell-free infectivity is released into the culture supernatant [5]. The enhancement of infectivity by cell–cell contact has been suggested to reflect the proximal coupling of virus assembly and entry machineries [6]–[9]. Indeed, morphological analyses have revealed HIV and HTLV-1 antigens clustering at cell–cell contact zones between antigen-presenting cells and T cells, as well as between infected and uninfected T cells [6],[10]–[13]. These cell–cell contacts are specifically enriched in microtubules, actin, and adhesion factors, and are designated as “virological” or “infectious” synapses due to their resemblance to the immunological synapse [10]–[12]. In addition to broad synaptic contacts, thin filopodial connections called cytonemes or nanotubes are utilized by retroviruses for the purpose of cell–cell spread [14]–[17]. Importantly, live-cell imaging has confirmed the direct transfer of retroviruses from one cell to another via both, thin filopodial connections and broad virological synapses [14],[18].

Here, we have applied live four-dimensional (4D) imaging in order to dissect the sequential stages of retroviral assembly, release, and transmission in real time for the model retrovirus murine leukemia virus (MLV). Our data reveal that after the establishment of contacts between infected and uninfected cells, the majority of virus particle assembly is initiated at sites of cell–cell contact. This bias in the site of virus production did not reflect any changes in particle assembly kinetics. Instead, contact-polarized assembly was dependent on signaling from the cytoplasmic tail of viral Env. In sum, we provide evidence that the initiation of retroviral assembly is directed towards infectious cell–cell interfaces, and identify the cytoplasmic tail of Env as a critical viral determinant for efficient intercellular spread.

## Results

**Figure 1:**
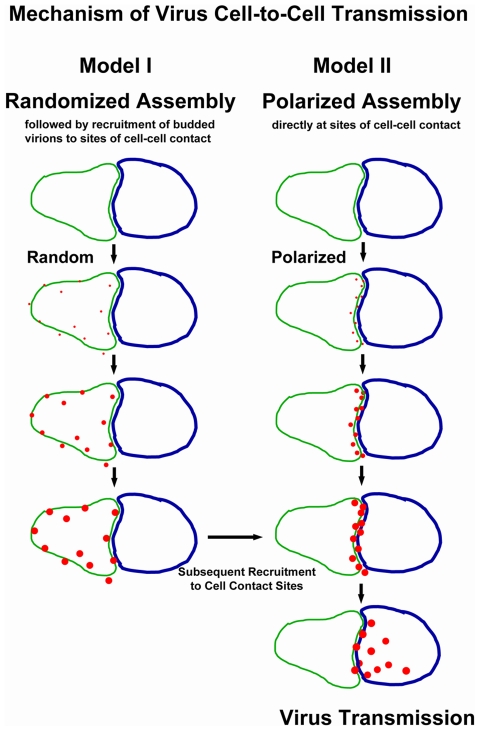
Models of virus cell-to-cell transmission. Two models have been proposed to explain the observed recruitment of retroviruses to sites of cell–cell contact. In Model I, virus assembly initiates randomly at the surface of virus-producing cell. Once assembly is completed, virus particles are subsequently recruited to sites of cell–cell contact, followed by the spreading of the infection to the target cell. In Model II, virus assembly is initiated preferentially at cell–cell contact sites, and nascent virus spreads to the target cells after the assembly is completed.

Viral components and fully assembled virions are known to accumulate at sites of contact between infected and uninfected cells, but the underlying details of this polarization are not well understood. According to one model, virus assembly is initiated at random at the cell surface. Following completion of assembly, viral particles would subsequently be recruited towards sites of cell–cell contact [13],[19] (Figure 1, Model I). Alternatively, virus assembly may be polarized and specifically initiated at zones of cell–cell contact. Following completion of assembly, viruses would spread and infect neighboring cells [7] (Figure 1, Model II). In order to distinguish between these models, we visualized and quantified de novo assembly of MLV in the absence and presence of cell–cell contact.

### Visualizing De Novo Assembly and Release of MLV in Living Cells

We used spinning disc confocal microscopy to visualize individual budding and cell-to-cell transmission events of retroviruses in three-dimensional space over time (4D). Compared to conventional confocal microscopes that contain a single pinhole, the Yokogawa SCU10 scan head used in our system contains about 20,000 microlenses that rotate at 1,800 rpm and allow the capture of confocal images at high speed and with little photobleaching. This allows the fast acquisition of *Z*-stacks of images over a long period of time, thereby recording the spatial information (3D) over time (4D). The 4D imaging allowed us to monitor the dynamics of virus assembly and release, as well as to follow the cell-to-cell transmission of viral particles. For these studies, we monitored the assembly and spread of the model retrovirus MLV because it allowed us to perform precise single-particle tracking (Figure 1) [14]. We have also made attempts to apply single-particle tracking to the transmission of HIV-1, but these have been impeded by the greater tendency of HIV-1 particles to aggregate at sites of cell–cell contact into big button or ring-shaped clumps of Gag punctae (unpublished data) [17],[18].

**Figure 2:**
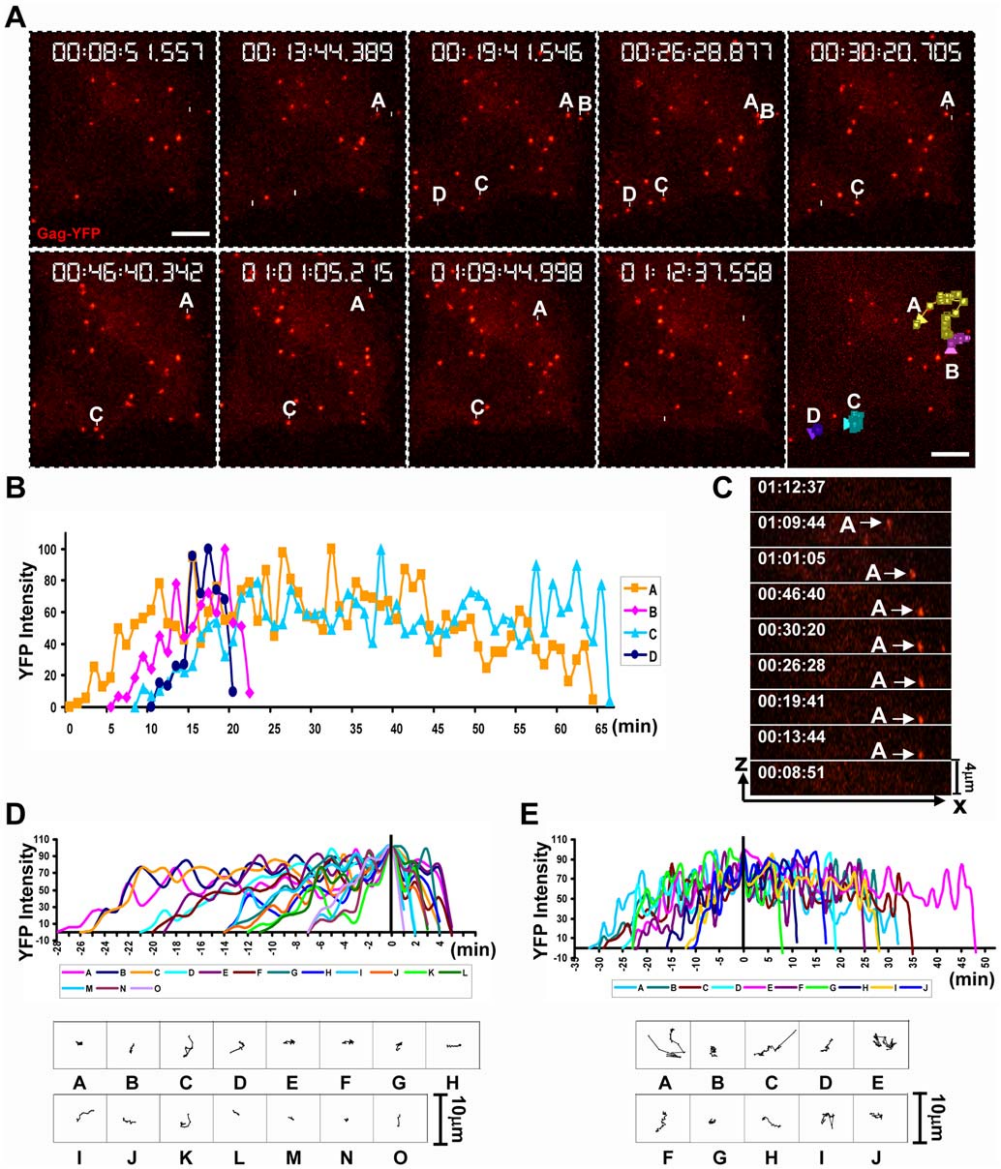
De novo assembly of murine leukemia virus in living cells. (A) Selective frames from Video S1 illustrate de novo appearance and release of Gag-YFP–labeled MLV particles (red) in HEK293 cells. Four representative particles were labeled A–D. The last panel represents the single-particle tracking analysis for these particles. The size bar corresponds to 10 µm. (B) Quantitative analysis of fluorescence intensity for particles A–D shown in (A) over time. (C) *XZ* presentation of particle A from (A) over time. (D and E) Single-particle tracking for viral particles that are either released shortly after reaching maximum intensity (D) or continued to stay associated with the plasma membrane prior to release (E). *XY* tracks for all particles are given below the graphs.

We first tested the ability of 4D imaging to detect de novo MLV assembly in the absence of target cells. HEK293 cells were transfected with plasmids encoding the viral components MLV GagPol, Gag-YFP, Env, and genome [20]. A GagPol to Gag-YFP ratio of 10∶1 allowed the production of fully infectious fluorescently labeled MLV viruses [20],[21]. Six hours following transfection, we identified cells that displayed a few YFP-positive punctae and monitored them by spinning disc confocal microscopy (Figure 2A). We detected the appearance of single fluorescent punctae that gradually intensified and then abruptly disappeared or underwent diffusive movement along the plasma membrane (Figure 2A, Video S1). Fluorescent punctae were tracked from the time they appeared, and their fluorescence intensity and *XYZ* coordinates were measured over time. The analysis showed that the fluorescence intensity of these punctae increased from background level over time. Once maximum intensity was reached, the punctae either abruptly dropped to background level or plateau undergoing diffusive movement along the plasma membrane (Video S1). For example, fluorescent punctae B and D presented in Figure 2A disappeared shortly after reaching maximum intensity, while punctae A and C remained associated with the cell surface for approximately 30 min prior to their sudden disappearance (Figure 2B). The observed increase in intensity followed by either disappearance or diffusive movement along the cell surface is consistent with the interpretation that these events represent de novo assembly followed by release of viral particles. We defined the assembly time as the amount of time that each particle took to achieve maximum intensity from background levels. For example, for the particles presented in Figure 2A, the time of assembly varied from 8 to 30 min (Figure 2B). Interestingly, analysis of the spatial information indicated that some particles formed at the glass–plate interface, completed assembly, and then migrated to the dorsal face of the cell prior to their disappearance (shown for particle A in Figure 2C, Video S1).

Combined, 25 of the 35 particles (∼70%) monitored in these experiments disappeared during the time of imaging. Of these, 60% vanished shortly after completion of assembly (Figure 2D). The other 40% remained associated with the plasma membrane for extended lengths of time, often undergoing rapid movements prior to release (Figure 2E). Similar results were obtained for COS-1 cells (Figure S1). In sum, the detection of fluorescent punctae that grow in intensity before either abruptly disappearing or undergoing diffusive movement along the plasma membrane is consistent with the interpretation that 4D imaging can detect individual retroviral budding events.

### Contact-Dependent Transmission of MLV in Cocultures of Virus-Producing Cells and Target Cells

**Figure 3:**
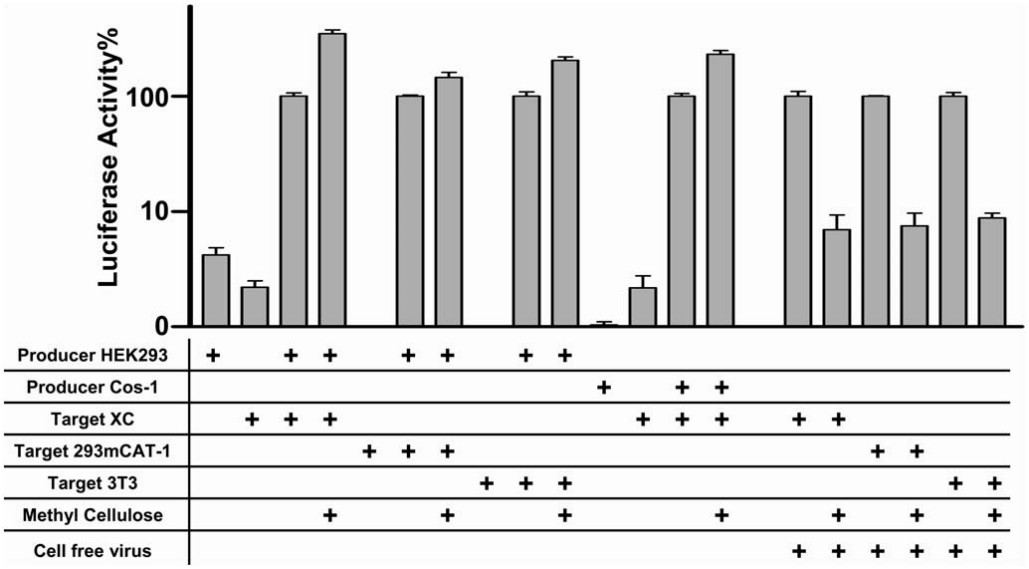
MLV transmission is contact-dependent. Producer cells (HEK293, Cos-1 cells) expressing an intron-regulated MLV luciferase reporter inLuc, MLV GagPol, and MLV Env were cocultured with target cells (XC, NIH 3T3, HEK293 cells stably expressing mCAT1) in the presence or absence of 1% methyl cellulose as indicated, and the resulting luciferase activity originating from expression in infected target cells was measured. The ability of 1% methyl cellulose to block infection of target cells by cell-free virus was tested to the right.

In order to study MLV cell-to-cell transmission in cell culture, we explored whether the virus spreads by a contact-dependent mechanism or whether cell-free virus dominates viral transmission. To distinguish between either modes, we cocultured infected and uninfected cells in a viscous 1% methyl cellulose solution previously demonstrated to slow the diffusion of large particles such as viruses [22]. We applied a quantitative assay that is based on an intron-regulated MLV luciferase reporter (inLuc), in which the expression of luciferase is prevented in producer cells and restricted to newly infected target cells (D. Mazurov, G. Heidecker, P. A. Lloyd, D. Derse, unpublished data). Interestingly, whereas 1% methyl-cellulose completely blocked infection with cell-free virus, the spread of MLV infectivity in cocultures of virus-producer cells and uninfected target cells was unaffected (Figure 3). The resistance of viral spread to 1% methyl cellulose was independent of the cell type used and was observed for transmission between the producer HEK293 and COS-1 cells and target cells such as rat XC, NIH3T3, and HEK293 cells expressing the MLV receptor mCAT1. Thus, despite the ability of MLV to be released into the medium (Figure 2), these results suggested that the predominant mode of MLV spread was through direct cell–cell transmission at physical interfaces (Figure 3).

### Visualizing De Novo Virus Assembly and Cell-to-Cell Transmission in Cocultures of Producer and Target Cells

**Figure 4:**
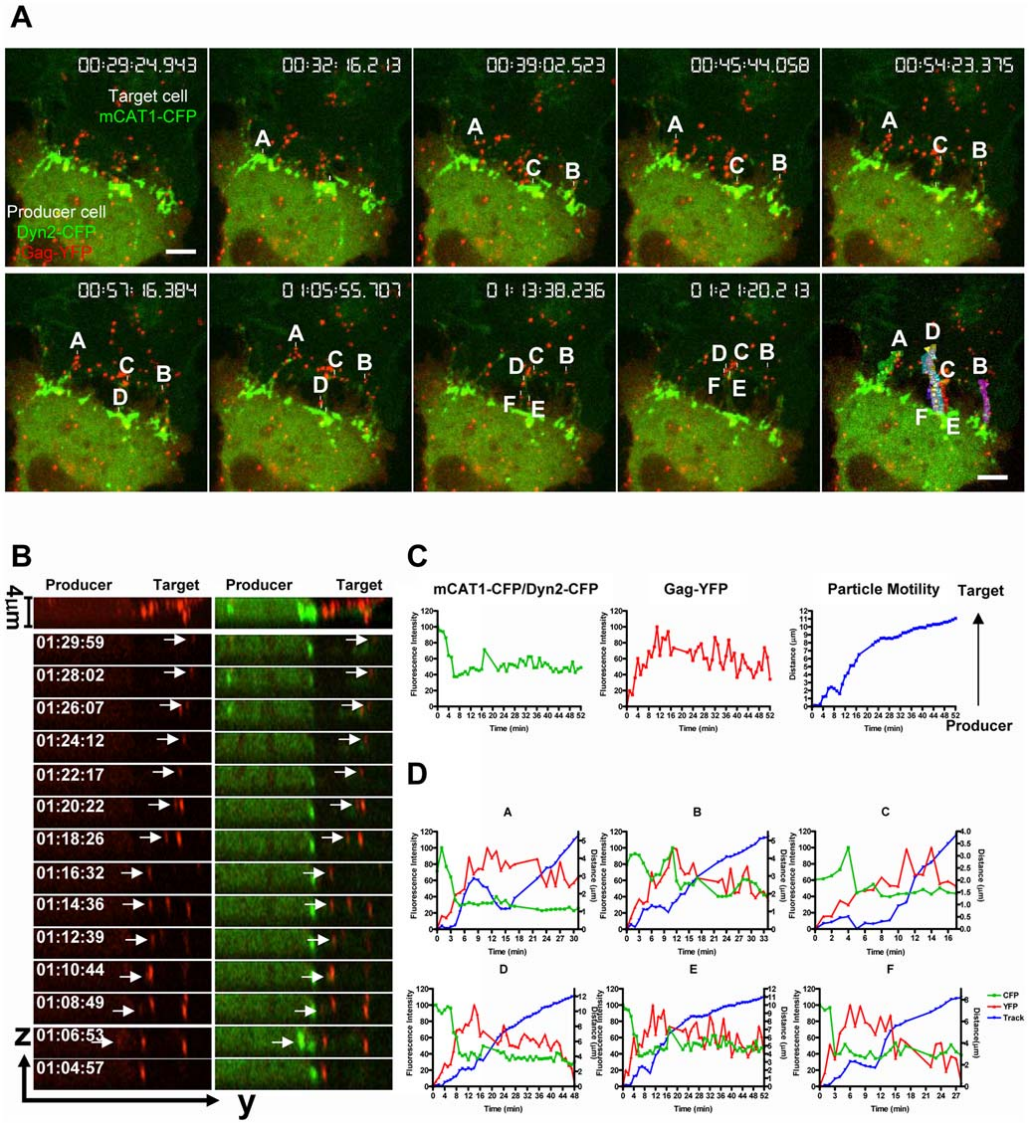
Assembly followed by transmission of MLV from virus-producing cells to target cells. (A) Selective frames from Video S2 monitoring the assembly of MLV particles (Gag-YFP, red) in HEK293 cells expressing dynamin2-CFP (green) followed by the transmission to target XC cells expressing receptor mCAT1-CFP (green). Six representative particles were labeled A–F. The last panel represents single-particle tracking analysis for these particles. The size bar corresponds to 10 µm. (B) Display of the *YZ* movement of particle E shown in (A). (C) Single-particle tracking analysis of mCAT1/dynamin2-CFP (green), Gag-YFP (red), and particle motility (blue) from producer to target cell of particle E over time. (D) Analysis as in (C) for particles A–F.

**Figure 5:**
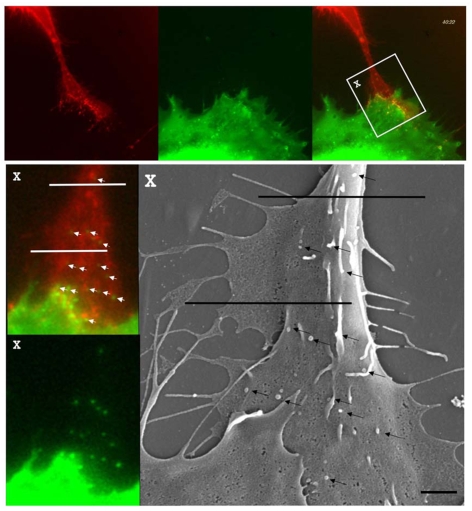
Fluorescent punctae correlate to single viral particles. COS-1 cells expressing MLV provirus and Gag-CFP (green) in contact with XC-mCAT1-YFP (red) were imaged by time-lapse microscopy. Following the detection of virus cell-to-cell transmission, the cells were fixed and processed for scanning electron microscopy. An edged in grid was used to re-identify region X in the FEI ESEM scanning electron microscope. Two black bars were introduced for orientations in the correlative images. Correlating fluorescence and SEM identifiable viral particles are indicated by white arrows (left) and black arrows (right). The size bar in the lower right corresponds to 1 µm.

We next applied these experimental conditions to monitor virus assembly in the context of cell-to-cell transmission. In addition to viral components, we cotransfected virus producer cells to express dynamin2-CFP, a marker protein that accumulates at cell–cell contacts [14]. Contact zones form specifically between Env and receptor expressing cells [14]. They are characterized by dynamin-containing endocytic areas where target cell membranes are anchored in the infected cell (Figure S2) [14]. Although dynamin2, expressed in the producer cell, has been implicated in the infectivity of HIV [23], transiently expressed wild-type or dominant-negative dynamin2-CFP did not affect the efficiency of virus cell-to-cell transmission, but facilitated the easy identification of cell–cell contacts (Figure S3). XC cells expressing a CFP-tagged version of the MLV receptor mCAT1 (mCAT1-CFP) were used as target cells [24]. XC cells were chosen because they exhibit a spread and dynamic peripheral actin cytoskeleton that assists visualization of distinct structural features at the cell–cell interface [14]. Five hours posttransfection, we initiated coculture of producer cells generating YFP-labeled MLV and target cells. Following 1 h of coculture, the accumulation of dynamin2-CFP and receptor-CFP molecules allowed us to clearly identify cell–cell contacts between producer and target cells (green). Strikingly, we observed a large number of MLV particles (red) emerging from the region of cell–cell contact (green) (Figure 4A, Video S2). Spatial analysis demonstrated that particles were formed at the edge of the producer cell contacting the target cell and then moved up towards the cell body of the target cell (shown for particle E in Figure 4B, Video S2). Correlative fluorescence and scanning microscopy confirmed that all observed fluorescent punctae correlated to single 100–150-nm viral particles (Figure 5) [25]. Single-particle tracking was used to identify de novo virus assembly events in virus-producing cells. These particles were then tracked over time, and their YFP (red) and CFP (green) fluorescence intensity as well as *XYZ* coordinates were measured (Figure 4C for particle E, and Figure 4D). The motility (blue) of each fluorescent spot was determined using the distance traveled between consecutive *XYZ* coordinates. Such an analysis revealed that assembly of viral particles (red) was specifically initiated in adhesive zones characterized by an accumulation of receptor and dynamin (green) (Figure 4C for particle E, and Figure 4D). Following completion of assembly, most particles were released from producer cells to migrate towards the target cell body (Figure 4C and 4D).

### Retroviral Assembly Is Directed towards Sites of Cell–Cell Contact

**Figure 6:**
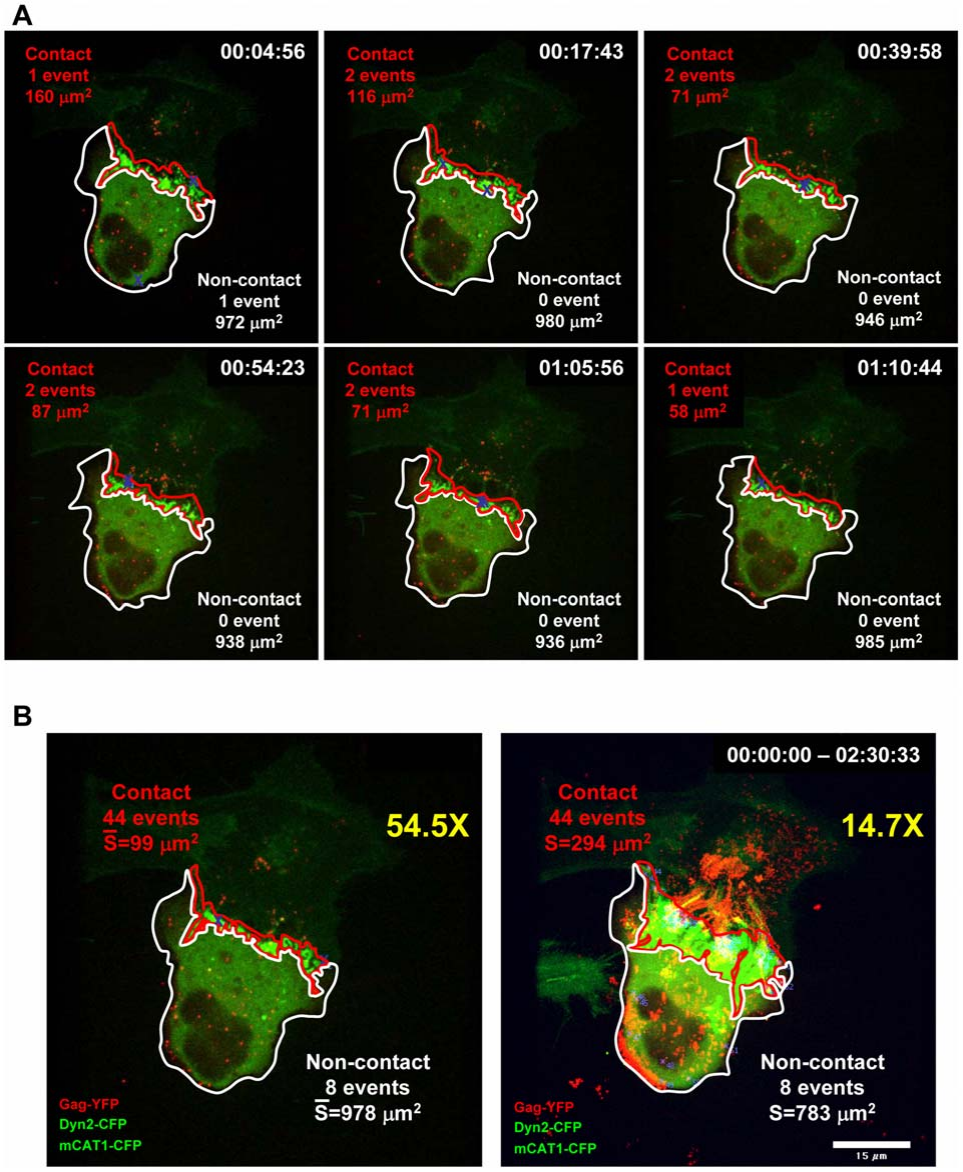
Quantification of MLV assembly events in and outside of the contact zone. (A) Six representative frames of a 157-frame video when an initiation of de novo virus assembly (blue cross) was detected. See Video S3 and Table S1 for the full analysis. The accumulation of dynamin2 and receptor-CFP was then used to define the contact zone (red line) in each frame. Homogeneous dynamin2 expression and Gag-YFP in the producer cell was used to mark the remaining plasma membrane surface outside the contact zone (white line). (B) Left panel. The analysis described in (A) identified 44 assembly events in the contact zone and eight outside the contact zone. To calculate the overall assembly frequency per surface unit (in square micrometers), the number of assembly events observed in either zone was divided by their average surface area. The resulting fold enhancement of MLV assembly at sites of cell–cell contact over the remaining plasma membrane is presented at the top right (54.5×). The underlying image used to illustrate this time-resolved analysis represents time point 00:19:43 of Video S3. Right panel. An alternative and simplified approach to define the fold enhancement was based on the accumulative merged image of all 157 frames of Video S3. Due to the dynamics of the contact zones, this approach results the definition of a broader contact zone thereby reducing the fold enhancement observed at sites of cell-cell contact (14.7×). For detailed analysis see Table S1. Size bars correspond to 15 µm.

**Figure 7:**
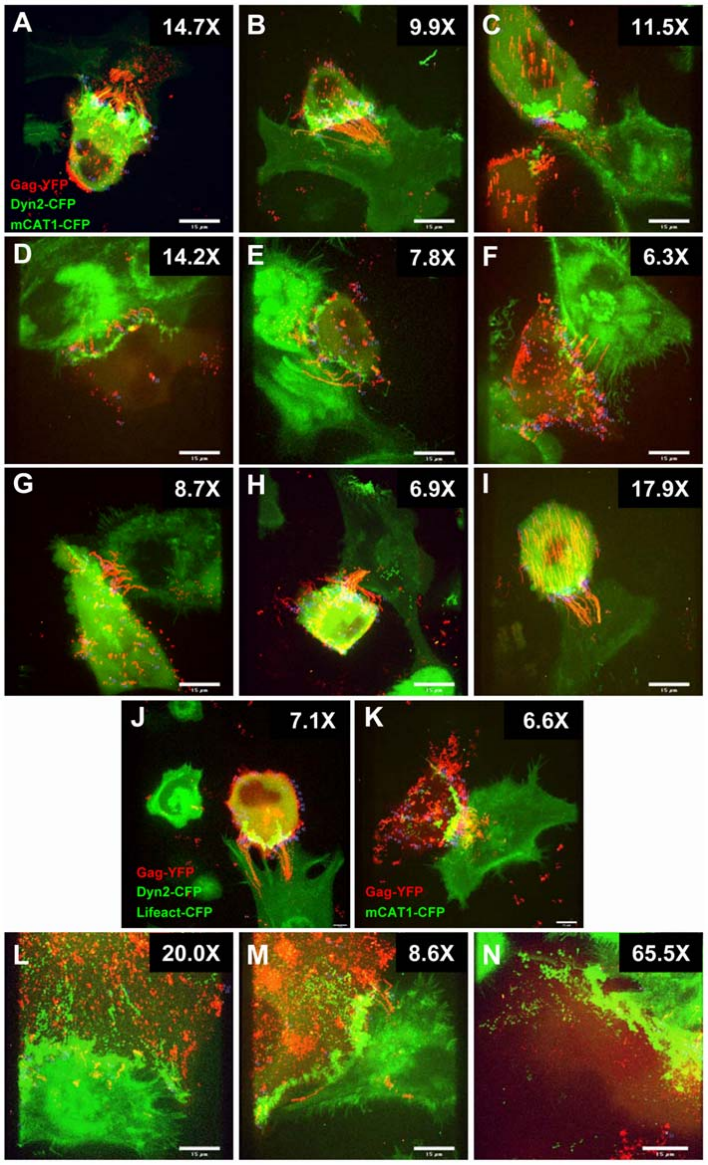
MLV assembly is directed towards sites of cell–cell contact. (A–I) Analysis of nine representative time-lapse videos monitoring MLV assembly in HEK293 cells in contact with XC target cells as described in Figure 6B. The individual panels represent the merged images of all frames of each video. Single-particle tracking was applied to identify all de novo assembly events and to calculate assembly frequency within and outside the cell–cell contact zone. The resulting fold enhancement of MLV assembly at sites of cell–cell contact over the remaining plasma membrane is presented at the top right of each panel. For detailed analysis, see Table S2. For comparison, (A) depicts the analysis from Figure 6B. The corresponding videos for (A, B, and C) are Videos S3, S4, and S5, respectively. (J) An analysis as in (A) was performed for cocultures between a MLV-producing HEK293 cell that expresses dynamin2-CFP to label the cell–cell contact and a XC target cell expressing actin-binding Lifeact-CFP. The corresponding video for panel (J) is Video S6. (K) An analysis as in (A) was performed for cocultures between a MLV-producing HEK293 cells and a HEK293 cell expressing mCAT1-CFP. The corresponding video for panel (K) is Video S7. (L–N) An analysis as in (A) was performed for cocultures between MLV-producing Cos-1 cells and XC target cells. The corresponding video for (N) is Video S8. Size bars correspond to 15 µm.

Being able to reliably detect de novo assembly events, we next asked whether the assembly events occurred “in” or “out” of cell–cell contact zones. Towards this end, we first identified all de novo MLV assembly events in virus-producing cells (blue crosses in Figures 6 and 7 and Video S3). Then, we defined contact zones in the virus-producing cell as the region enriched in dynamin2-CFP and receptor mCAT1-CFP. Because the contact zones are dynamic over time, the surface area of the contact zone (red line) as well as the noncontact zone (white line) were measured for the 37 time points when de novo virus assembly events were detected (Figure 6A, Table S1). This analysis revealed 44 assembly events in the contact zone and eight outside of the contact zone (Video S3). To calculate the overall assembly frequency per surface unit (in square micrometers), the number of assembly events observed in either zone was divided by their average surface area in all the 37 frames with assembly events (Figure 6B, left panel, Table S1). The ratio of the normalized assembly frequency occurring in or out of the contact zone served as an indicator for the fold enhancement of assembly at contact zone. For the cell–cell contact shown in Figure 6, this analysis revealed a striking 54.5-fold enhancement of MLV assembly in zones of cell–cell contact (Figure 6B, left panel, Table S1). A simplified approach whereby all frames of the time-lapse video were overlaid into a single image to define a larger contact zone (Figure 6B, right panel) still revealed a 14.7-fold enhancement (Figure 6B, Table S1). Although the non–time-resolved analysis clearly underestimated the stimulation of assembly at sites of cell–cell contact, it proved to be a rapid and reliable method that allowed the quantification of a large set of time-lapse videos. We applied this method to analyze additional contacts between HEK293 cell and XC cell expressing mCAT1-CFP. The observed stimulation of assembly at sites of cell–cell contact varied between 6- and 18-fold and averaged 11-fold for nine representative contacts (Figure 7A–7I, Table S2, Videos S3, S4, and S5). A similar enhancement of assembly was observed for cocultures of MLV producing COS-1 cells and XC target cells (Figure 7L–7N, Table S2, Video S8) as well as for coculture of MLV producing HEK293 cells and HEK293 cells expressing mCAT1-CFP (Figure 7K, Table S2, Video S7). This enhancement of assembly at cell–cell contact was independent of expression of mCAT1-CFP in target cells or dynamin2-CFP in producer cells (Figure 7J and 7K, Table S2, Videos S6 and S7). Thus, 4D imaging of MLV assembly in the absence and presence of cell–cell contact revealed a striking enhancement of MLV assembly at sites of cell–cell contact.

### MLV Assembly Kinetics Are Similar in the Absence or Presence of Cell–Cell Contact

**Figure 8:**
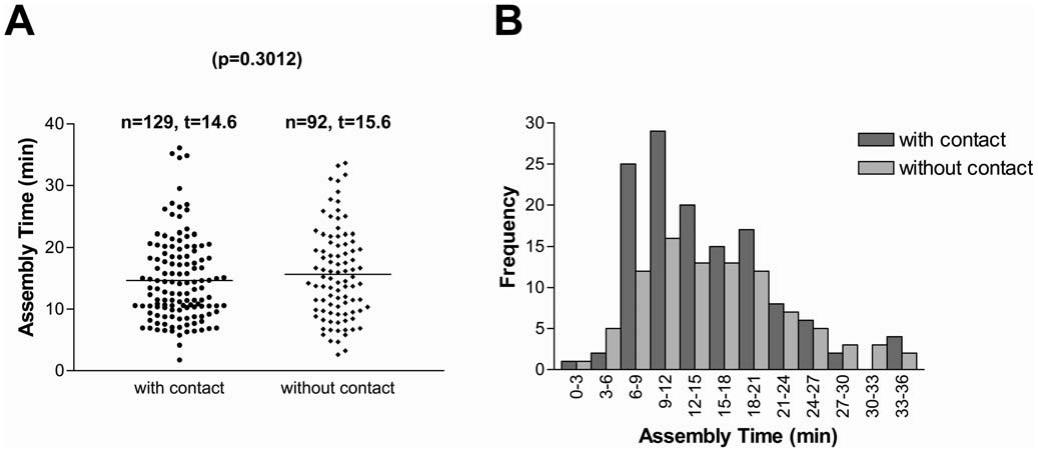
Virus assembly is not accelerated by cell–cell contact. (A) The assembly time (minutes), defined as the time that it takes a particle to reach maximum intensity from background levels, is displayed for 129 assembly events in zones of cell–cell contact (with contact) and for 92 assembly events in the absence of cell–cell contact (without contact). The average assembly times (*t*) are given above the graphs. (B) Histograph of assembly time for particles assembled in the presence (with contact) and absence (without contact) of cell–cell contact.

To understand the nature of enhancement of virus assembly at sites of cell–cell contact, we first tested the possibility that assembly is accelerated by contact. Comparative analysis of MLV assembly events in the presence or absence of cell–cell contact revealed that the average MLV assembly time was similar, 14.6 and 15.6 min, respectively (Figure 8A). The *p*-value of 0.3012 indicated that both values did not significantly differ. The distribution of assembly time for MLV assembly events in the presence or absence of cell–cell contact was also similar, with the most frequently observed assembly time ranged between 9 and 12 min (Figure 8B). Thus, the process of virus assembly is not accelerated at sites of cell–cell contact and proceeds within 14–15 min irrespective of the location.

### Assembly-Deficient MLV Gag Is Recruited to Sites of Cell–Cell Contact

**Figure 9:**
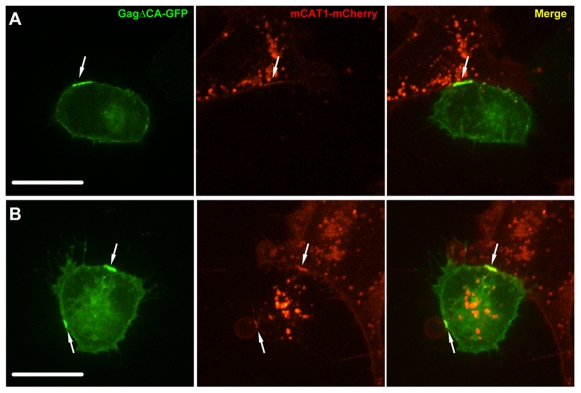
Monomeric Gag is recruited to cell–cell contact. (A,B) HEK293 cells transfected with full-length MLV provirus lacking capsid domain (MLVΔCA-GFP, green) were cocultured with XC target cells expressing mCAT1-mCherry (red). MLVΔCA-GFP is recruited to sites of cell–cell contact with accumulation of receptor mCAT1-mCherry. Size bars correspond to 17 µm.

We next asked the question whether a local increase in Gag concentration leads to the enhancement of assembly at sites of cell–cell contact. To test this possibility, HEK293 cells were transfected with an assembly-deficient MLV provirus lacking the capsid domain (MLVΔCA-GFP). This Gag mutant exists intracellularly as a monomer and does not form virus particles. Interestingly, MLVΔCA-GFP was recruited to sites of cell–cell contact (Figure 9). These results indicate that the local concentration of monomeric Gag is increased at contact zones. These data, taken together with our earlier observation that virus assembly is specifically initiated in contact zones (Figures 4, 6, and 7), suggest that nucleation of assembly, the rate-limiting step of many polymerization reactions, is enhanced at sites of cell–cell contact.

### Polarized Assembly of MLV towards Sites of Cell–Cell Contact Requires an Intact Env Cytoplasmic Tail

**Figure 10:**
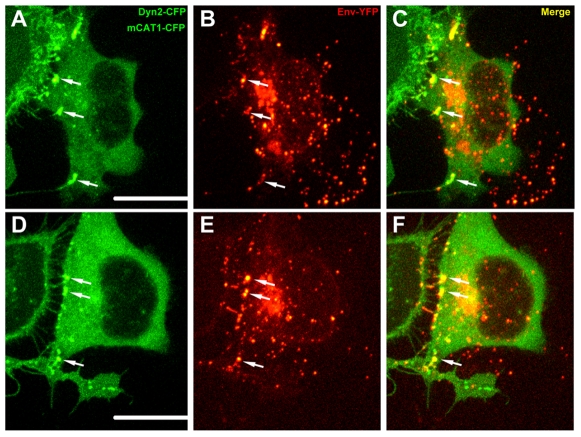
Env localizes to sites where target cell membranes are anchored in infected cells. (A-F) HEK293 cells expressing MLV GagPol, genome, MLV Env-YFP (red), and dynamin2-CFP (green) were cocultured with XC target cells expressing mCAT1-CFP (green). MLV Env-YFP efficiently localized to sites of cell–cell contact in addition to being incorporated into punctate virions. Size bars correspond to 15 µm.

**Figure 11:**
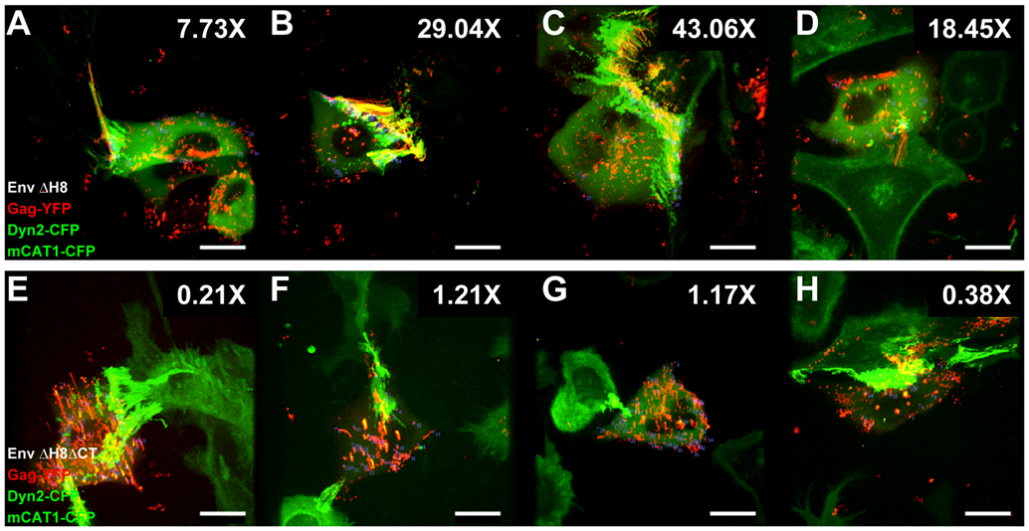
Polarized assembly depends on the presence of the cytoplasmic tail of Env. (A–D) An experiment as in Figure 7 was performed with MLV Env lacking histidine 8 (Env ΔH8). The fold enhancement of MLV assembly at sites of contact is given at the top right of each panel. The corresponding video for (D) is Video S9. (E–H) An experiment as in (A–D) was performed for mutant Env lacking the cytoplasmic tail (Env ΔH8ΔCT). The corresponding video for (F) is Video S10. Size bars correspond to 15 µm.

Polarization in other biological systems is governed by adhesion proteins that redirect protein sorting towards sites of cell–cell contact to establish polarity [26]. Intriguingly, during virus cell-to-cell transmission, the establishment of cell–cell contact is driven by a high-affinity interaction between viral Env glycoprotein and receptor mCAT1 [14]. Consequently, Env accumulates at sites of cell–cell contact (Figure 10). Given that the cytoplasmic tail domains of transmembrane adhesion proteins can contribute to establishing cellular polarity, we deleted the cytoplasmic tail of Env in order to determine whether it plays a role in the polarization of assembly. MLV Env glycoproteins are single-pass transmembrane proteins, and their cytoplasmic tails have been shown to regulate Env fusogenicity [27]–[29]. Because C-tail deletion can lead to a high degree of cell–cell fusion in infected cultures, we deleted the histidine residue at position 8 of Env (Env ΔH8), known to suppress Env fusogenicity without compromising receptor binding [30],[31]. The formation of cell–cell contacts and polarization of virus assembly to contact sites appeared unaltered for EnvΔH8 (Figure 11A, Table S3, Video S9). In contrast, polarized assembly was completely abolished for Env ΔH8 lacking the cytoplasmic tail (Env ΔH8ΔCT) despite efficient formation of cell–cell contacts that were indistinguishable from wild-type Env (Figure 11B, Table S3, Video S10). Although we cannot exclude the possibility that contact zone dynamics are altered, these data suggest a model whereby direct or indirect signaling via the cytoplasmic tail of Env directs Gag trafficking to sites of cell–cell contact.

### Polarized Assembly during Repeated Rounds of MLV Cell-to-Cell Transmission

**Figure 12:**
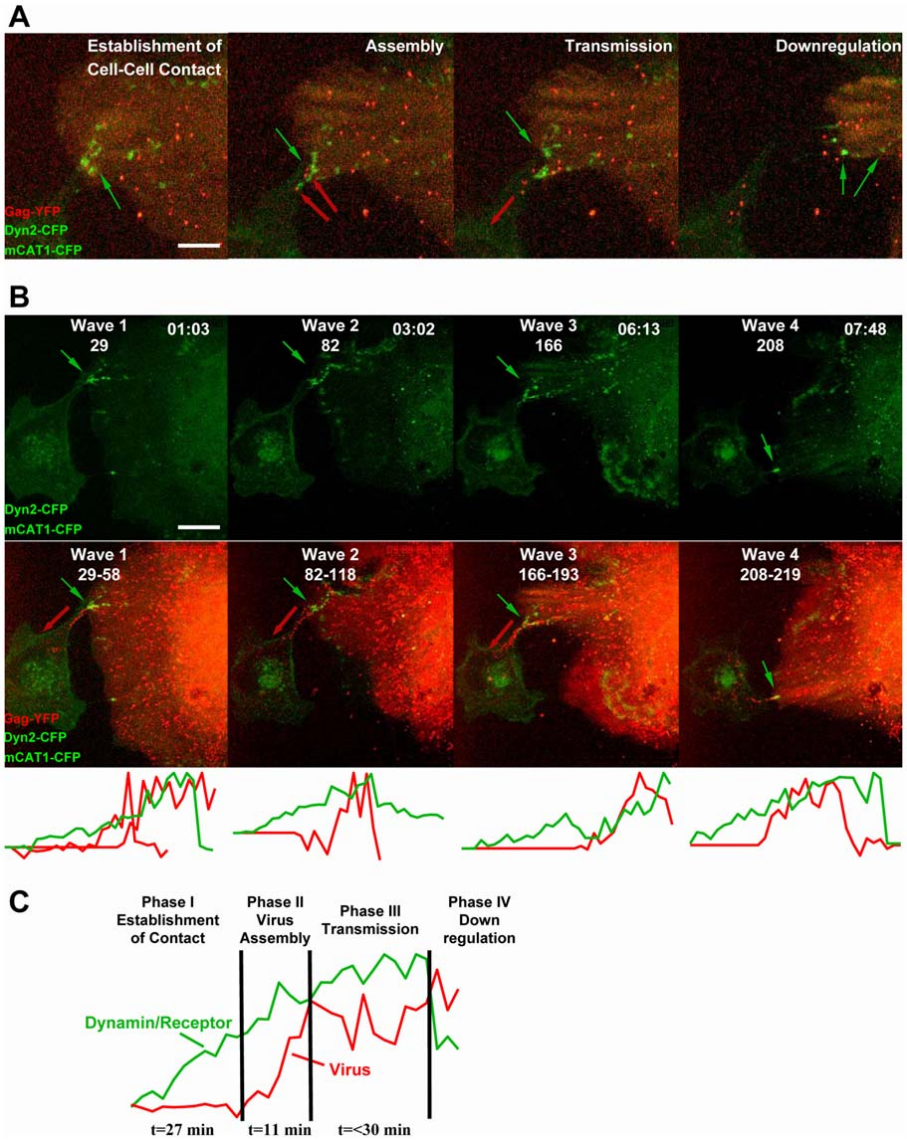
Polarized assembly in the context of four phases of cell-to-cell transmission. (A) COS-1 cells expressing dynamin2-CFP (green) and producing fluorescently labeled MLV (Gag-YFP, red) were cocultured with XC cells expressing the MLV receptor mCAT1-CFP (green). Time-lapse microscopy over the period of 8.5 h revealed four “waves” of cell-to-cell transmission (Videos S11 and S12). Each wave contained four phases, the establishment of cell–cell contact, characterized by the accumulation of dynamin2-CFP and receptor mCAT1-CFP at sites of cell–cell contact (green arrow), virus assembly out of cell–cell contact sites (red arrows), cell-to-cell transmission of viral particles (red arrow), and down-regulation of the contact. The four phases presented here correspond to wave 3 in (B). (B) Graphical presentation and quantitative analysis of the four observed transmission events. The upper panels present the four time points (h∶min) of each “wave” when cell–cell contact was established prior to the induction of virus assembly. The lower panels represent merged frames of the time period of Video S12 that describes the four virus assembly and transmission events (frames 29–58, 82–118, 166–193, and 208–219 of Video S12). Finally, the quantitative analysis of these four transmission events for the adhesion markers receptor/dynamin2 (green) and viral particles (red) is given below the images. (C) The average composite of the four analyses shown in (B) illustrate the four phases of cell-to-cell transmission. Size bars correspond to 15 µm.

Long-term imaging experiments of several hours allowed us to monitor polarized assembly in the context of the formation and dissociation of cell–cell contacts. In this case, when imaging was initiated, we could readily observe completely assembled viral particles randomly located at the plasma membrane of the producer cells. However, in response to the establishment of cell–cell contact, we observed that assembly was coordinated with cell-to-cell transmission. We observed that cell-to-cell transmission proceeds in four phases (Videos S11 and S12). A representative cell–cell contact established between a virus-producing Cos-1 cell and the receptor expressing target cell is presented in Figure 12A (Videos S11 and S12). Both Dynamin2-CFP and mCAT1-CFP (green) accumulated together at sites of contact during Phase I (Figure 12A). In Phase II, de novo MLV assembly (Gag-YFP, red) was induced at contact zones, and numerous bright particles were generated. The assembly frequency at these sites of cell–cell contact was elevated as compared to the occasionally observed assembly of few viral particles outside of contact zones. During Phase III, viruses were released and moved along filopodial bridges towards the target cell (Figure 12A). Finally, in Phase IV, virus transmission was stopped by the apparent down-regulation of receptor/dynamin complexes at contacts, resulting in cell separation (Figure 12A).

Over the period of 8.5 h, we observed four consecutive “waves” of contact, polarized assembly, virus transmission, and cell separation (Figure 12B, Videos S11 and S12). Quantitative analysis of the CFP-labeled receptor/dynamin2 (green) and Gag-YFP fluorescence (red) for each wave indicated that the establishment of contact preceded virus assembly (Figure 12B, Video S12). The average composite of these four transmission events allowed us to generalize our observations (Figure 12C). It took approximately 30 min to establish cell–cell contact before the first virus assembled. Assembly of individual viruses proceeded in approximately 10 min. In the subsequent transmission phase, which lasted approximately 30 min, additional viruses assembled at the contact site and moved towards target cells. Finally, transmission was terminated due to contact down-regulation (Figure 12C). In this system, the establishment and maintenance of cell–cell contact lasted approximately 1 h, whereas assembly was relatively swift, proceeding in approximately 10 min. Taken together, long-term imaging demonstrated that virus assembly at the plasma membrane of infected cells can be polarized in response to the establishment of cell–cell contact, reinforcing the notion of a contact-induced switch from random to polarized assembly.

## Discussion

It has long been known that retroviral spreading is more efficient when cells can physically interact with each other [1]–[4],[8]. Applying 4D imaging and single-particle tracking, we have demonstrated that the murine leukemia virus can redirect virus assembly to sites of cell–cell contact for transmission to neighboring cells. As such, our results support a model of polarized assembly as the primary cause for the accumulation of viral particles at zones of cell–cell contact (Model II in Figure 1). Our data contribute to the emerging picture that several steps of the viral life cycle are efficiently coordinated at sites of cell–cell contact. Future work will reveal to what extent our model applies to other viruses and experimental conditions.

Our work is based on the ability of spinning disc confocal microscopy to detect de novo assembly and monitor the subsequent spatial movement of completely assembled particles. Applying a cautious definition of contact zones, our visual approach revealed an approximately 10-fold enhancement of virus assembly at sites of cell–cell contact. In the absence of cell–cell contact, particle release from producer cells into the culture supernatant was observed, consistent with the production of cell-free virus. Yet, in the context of coculture, MLV assembly was strongly directed towards sites of cell–cell contact, followed by efficient transmission to target cells. These data indicated that although assembly occurs randomly at plasma membrane, assembly becomes polarized following the establishment of cell–cell contact.

In an effort to understand the mechanism of the enhancement of assembly at sites of cell–cell contact, we observed no acceleration of assembly. On average, the assembly time observed for MLV in HEK293 cells was approximately 15 min, slower in comparison to the approximately 8 min observed for HIV in HeLa cells [32]. MLV assembly was even slower in COS-1 cells, averaging 20.2 min for 79 events, suggesting that assembly time varies depending on the cell type (Figure S1, unpublished data). Future experiments carried out in the same cell type in parallel are required to address the observed differences between HIV and MLV.

Although virus assembly per se was not accelerated at the sites of cell–cell contact, Gag proteins that drive virus particle assembly were recruited to cell–cell contacts. An elevation of Gag levels at contact sites may increase the frequency of nucleation, thereby enhancing virus assembly. The polarization of assembly required the cytoplasmic tail of the viral Env glycoprotein. Evidence for a communication between the cytoplasmic tail of retroviral Env and Gag proteins has been reported [33]–[36]. Env expressed in polarized cells such as MDCK cells and neurons can relocalize Gag [37]–[39]. In this work, we demonstrate that the establishment of cell–cell adhesion following Env/receptor interactions can break symmetry and establish polarity in otherwise nonpolarized fibroblasts. Future work is needed to understand whether the communication between Env and Gag is direct or indirect.

Our results reinforce similarities between virological and biological synapses in that the establishment of cell–cell adhesion is followed by polarization and the directed delivery of ligands towards sites of cell–cell contact [26]. Our data suggest that the MLV Env glycoprotein functions analogously to a cellular adhesion protein that establishes cell–cell contact and polarizes cells. Intriguingly, once MLV Env is packaged into virions, during or soon after virus budding, the cytoplasmic tail is cleaved off by the viral protease [40]–[42]. As such, the viral protease transforms an adhesion protein into a highly fusogenic fusion protein to mediate virus-to-cell fusion. This mechanism represents yet another clever adaptation and utilization of cellular principles by viruses to favor efficient viral spreading.

## Materials and Methods

### Reagents and Cell Lines

Plasmid encoding MLV GagPol, MLV LTR-LacZ, MLV Gag-YFP, MLV Env-YFP, mCAT1-CFP, and dynamin2-CFP were described previously [14],[20]. Plasmids encoding mutant Friend MLV EnvΔH8 was a gift from J. Cunningham (Harvard Medical School, Boston, MA). The cytoplasmic tail of Env ΔH8 was truncated at the viral protease cleavage site by PCR-based mutagenesis to generate MLV Env ΔH8ΔCT. This truncation has also been designated R peptide minus mutant [28],[42]. CA and Pol coding regions were deleted, and the GFP coding region was fused to C-terminal of NC Full-length Friend MLV genome to generate a mutant provirus that expresses GFP-fused deltaCA Gag as well as Env. HEK293, COS-1, and NIH 3T3 cells were maintained in DMEM high glucose (Invitrogen) containing 10% FBS plus Pen/Strep/Glutamine. Rat XC sarcoma cells were grown in MEM (Invitrogen) with 10% FBS plus Pen/Strep/Glutamine. XC cells stably expressing mCAT1-CFP and HEK293 cells stably expressing mCAT1 were selected using G418 (Invitrogen) and twice FACS-sorted for mCAT1 surface expression. Monoclonal mouse anti-human dynamin antibody (BD Biosciences) was used to stain endogenous dynamin in virus-producing cells.

### Live-Cell Imaging

For live confocal imaging, virus-producing cells and target cells were cocultured in MatTek glass-bottom plates that were pretreated with 0.2 mg/ml fibronectin (Invitrogen) for 10 min at room temperature. To generate HEK293 cells producing fluorescently labeled MLV, cells were transfected in 24-well plates using 800 ng of total DNA (244 ng of MLV Env or MLV Env mutant, 254 ng of MLV GagPol, 26 ng of MLV Gag-YFP, 244 ng of MLV LTR-LacZ, and 32 ng of dynamin2-CFP) and 2 µl of Lipofectamine 2000 (Invitrogen) per well. To generate COS-1 cells producing fluorescently labeled MLV, cells were transfected in six-well plates using 1,200 ng of total DNA (366 ng of MLV Env, 381 ng of MLV GagPol, 39 ng of MLV Gag-YFP, 366 ng of MLV LTR-LacZ, and 48 ng of dynamin 2-CFP) and 3.6 µl of FuGene 6 reagent (Roche) per well. At 4 h post HEK293 transfection and 22 h post Cos-1 transfection, virus-producing cells were replated in fibronectin-coated MatTek plates. One hour later, XC cells expressing mCAT1-CFP were added to start coculture; 1 h post initiation of coculture, live imaging was performed using the 60× objective of a Volocity spinning disc confocal microscope equipped with an environmental chamber (LIVE CELL; Pathology Devices) and a Nikon Perfect Focus. We took advantage of the Perfect Focus to simultaneously image multiple cell–cell contacts over time. All time-lapse videos were edited using Volocity, Openlab software (Improvision/PerkinElmer) and ImageJ. Videos were saved for presentation in QuickTime format using Sorensen 3 compression for videos. Single-particle tracking of 986 particles, analyzed in this work, was performed using the Quantitation software package from Volocity (Improvision/PerkinElmer). Fluorescent punctae were identified and their YFP and CFP fluorescence intensity as well as XYZ coordinates determined over time. Additional analysis and data presentations were performed following the export of datasets into Microsoft Excel.

### Scanning Electron Microscopy

Correlative fluorescence and scanning electron microscopy was essentially as previously described [24]. Briefly, cells were cocultured on MatTek dishes carrying an etched grid for cell re-identification (MatTek). Immediately after live imaging, cells were fixed in 4% PFA, washed three times with PBS, and then returned to the wide-field fluorescence microscope. Samples were subsequently processed for scanning electron microscopy. Cells were fixed for 30 min with 2.5% glutaraldehyde/2% paraformaldehyde in 100 mM cacodylate buffer (pH 7.4), rinsed three times with 100 mM cacodylate buffer, and dehydrated through a graded ethanol series. After washing three times with hexamethyldisilazane (EMS), cells were dried for 5 min at 60°C and coated with platinum. The grid was used to re-identify regions of interest and the area analyzed using a FEI ESEM scanning electron microscope (Philips).

### Intron-Regulated Luciferase-Based MLV Transmission Assay

To measure MLV cell-to-cell transmission, we applied a quantitative assay that is based on an intron-regulated MLV luciferase reporter (inLuc), in which the expression of luciferase is prevented in producer cells and restricted to newly infected target cells (D. Mazurov, G. Heidecker, P. A. Lloyd, D. Derse, unpublished data). To generate virus-producing cells, HEK293 producer cells were transfected with plasmids encoding the MLV inLuc reporter, MLV Gag Pol, and MLV Env. HEK293 cells were transfected in 24-well plates using 800 ng of total DNA (266 ng of MLV Env, 267 ng of MLV GagPol, and 267 ng of MLV LTR-inLuc) and 2 µl of Lipofectamine 2000 (Invitrogen) per well. COS-1 cells were transfected in six-well plates using 1,200 ng of total DNA (MLV Env 400 ng, MLV GagPol 400 ng, and MLV LTR-inLuc 400 ng) and 3.6 µl of FuGene 6 reagent (Roche). At 6 h post HEK293 transfection and 24 h post COS-1 transfection, producer cells were cocultured with target cells at a 2∶1 ratio for 24 h in the absence or presence of 1% methyl cellulose. At the end of coculture, cells were lysed and the luciferase activity measured using a Berthold Technologies Centro LB960 Luminometer.

## Supporting Information

Figure S1 Visualizing MLV assembly and release in Cos-1 cells. (A) An experiment as in (Figure 2A and 2B) was performed in Cos-1 cells generating fluorescently labeled MLV (Gag-YFP, red). (B) Release of a particle from Cos-1 cells into the medium following completion of assembly.
(1.70 MB TIF)

Figure S2 Endogenous dynamin is recruited to sites where target cell membranes are anchored in infected cells. HEK293 cells expressing MLV genome, Env, GagPol, and Gag-YFP (green) were cocultured with XC target cells expressing mCAT1-CFP (blue). Cells were fixed and permeabilized at 3 h post coculture. Endogenous dynamin was stained with dynamin antibody and Alexa568 conjugated secondary antibody (red). Endogenous dynamin localized to sites of cell-cell contact where receptor and virus particles accumulated. Size bars correspond to 15 µm.
(0.42 MB TIF)

Figure S3 Expression of wild-type and dominant-negative dynamin2 does not affect MLV cell-to-cell transmission. A control experiment as in Figure 3 was performed to determine potential effects of dynamin2 expression on the efficiency of virus cell-to-cell transmission. Producer cells (HEK293 cells) expressing an intron-regulated MLV luciferase reporter inLuc, MLV GagPol, and MLV Env, as well as either wild-type dynamin2-CFP or K44A dynamin2-CFP or CFP control, were cocultured with target cells (XC, NIH 3T3, HEK293 cells stably expressing mCAT1). The luciferase activity originating from infection of target cells is presented.
(0.11 MB TIF)

Table S1 Calculation of the fold enhancement of MLV assembly at sites of cell-cell contact as presented in Figure 6. Single-particle tracking was applied to identify all de novo assembly events in the MLV-producing HEK293 cell cocultured with XC-expressing mCAT1-CFP in Video S3. Number of assembly events as well as the surface area of contact and the noncontact zones are listed for each frame when initiation of de novo assembly can be detected. The surface area of contact and the noncontact zones in the merged image of all the frames were also listed. To determine the assembly frequency in the absence or presence of cell contact, the number of assembly events observed inside or outside of contact zones was normalized to the respective surface area. For frame-by-frame analysis, average surface area was used, and for overlay analysis, surface area in the merged image was used. To obtain the fold enhancement of MLV assembly in zones of cell-cell contact, the assembly frequency in the presence of cell-cell contact was divided by the assembly frequency in the absence of contact.
(0.17 MB PDF)

Table S2 Calculation of the fold enhancement of MLV assembly at sites of cell-cell contact as presented in Figure 7. Single-particle tracking was applied to identify all de novo assembly events in MLV-producing cells (HEK293, COS-1 cells) cocultured with receptor-expressing target cells. To determine the assembly frequency in the absence or presence of cell contact, the number of assembly events observed inside or outside of contact zones was normalized to the respective surface area in the frame merged images. To obtain the fold enhancement of MLV assembly in zones of cell-cell contact, the assembly frequency in the presence of cell-cell contact was divided by the assembly frequency in the absence of contact. In addition, the table lists technical parameters of the underlying time-lapse videos such as the total imaging time and the frame time.
(0.16 MB PDF)

Table S3 Calculation of the fold enhancement of MLV assembly observed for cells expressing wild-type or mutant Env lacking the cytoplasmic tail as presented in Figure 11. A calculation as in Table S2 was performed for cells expressing Env carrying a histidine 8 deletion (Env ΔH8) and Env lacking a cytoplasmic tail in addition to the histidine 8 mutation (Env ΔH8ΔCT).
(0.17 MB PDF)

Video S1 Visualization of de novo assembly and release of MLV in living cells. A HEK293 cell generating MLV labeled with Gag-YFP (red) was monitored using spinning disc confocal microscopy over a period of 1 h and 12 min. A z-stack of 13 images covering 4 µm was acquired every 59 s. To generate the QuickTime video, the 13 frames were merged into a single image using Improvision Volocity and Openlab software. The last frame of the QuickTime video displays the merged image of all frames of the time-lapse video. The video is played at 10 frames/s. The four labeled particles correspond to the particles A–D presented in Figure 2A.
(1.72 MB MOV)

Video S2 Cell-to-cell transmission of virus particles towards target cells following completion of assembly. A HEK293 cell generating MLV labeled with Gag-YFP (red) and expressing dynamin2-CFP (green) in contact with a noninfected target XC cell expressing mCAT1-CFP (green) was monitored using spinning disc confocal microscopy. The cell-cell contact was imaged over a period of 1 h and 35 min. A z-stack of red and green images was taken every 60 s and merged into a single file to generate a QuickTime video. The video represents first the “red/green” sequence followed by the “red only” to facilitate the detection of de novo assembly events. At the end of each sequence, a single image is presented to display the merged image of all frames of the time-lapse video. The video is played at 10 frames/s. The six labeled particles correspond to particles A–F in Figure 4A.
(5.86 MB MOV)

Video S3 Identification of de novo assembly events. Video S3 represents a larger viewing field of the cell-cell contact displayed in Video S2. Blue crosses were used to label the sites where virus particles started to assemble during the imaging time. At the end of the sequence, a single image is presented to display the merged image of all frames of the time-lapse video. The video is played at four frames/s.
(8.90 MB MOV)

Video S4 Retroviral assembly is directed towards sites of cell-cell contact. An experiment as described for Video S3 for an additional cell-cell contact. The video is played at 10 frames/s. Video S4 represents the data source for the analysis presented in Figure 7B. Statistical data for this video can be found in Table S2.
(12.61 MB MOV)

Video S5 Retroviral assembly is directed towards sites of cell-cell contact. An experiment as described above for an additional cell-cell contact. The video is played at 10 frames/s. Video S5 corresponds to Figure 7C. Statistical data for this video can be found in Table S2.
(20.84 MB MOV)

Video S6 Retroviral assembly is directed towards sites of cell-cell contact. Time-lapse video monitoring the cell-cell interaction between a HEK293 cell producing MLV and XC target cell expressing Lifeact-CFP. The video is played at 10 frames/s. Video S6 corresponds to Figure 7J. Statistical data for this video can be found in Table S2.
(6.66 MB MOV)

Video S7 Retroviral assembly is directed towards sites of cell-cell contact. Time-lapse video monitoring the cell-cell interaction between a HEK293 cell producing MLV without coexpression of dynamin2-CFP and a HEK293 cell expressing mCAT1-CFP. The video is played at 10 frames/s. Video S7 corresponds to Figure 7K. Statistical data for this video can be found in Table S2.
(6.56 MB MOV)

Video S8 Retroviral assembly is directed towards sites of cell-cell contact. Time-lapse video monitoring the cell-cell interaction between a COS-1 cell producing MLV and XC target cells. The video is played at 5 frames/s. Video S8 represent the data source for the analysis presented in Figure 7N. Statistical data for this video can be found in Table S2.
(9.54 MB MOV)

Video S9 Env containing intact cytoplasmic tail directs assembly towards sites of cell-cell contact. Time-lapse analysis as described in Video S3 was performed for cells expressing MLV Env with a deletion of histidine 8. The statistical data for this video can be found in Table S3. The video is played at 10 frames/s and corresponds to the analysis shown in Figure 11D.
(9.44 MB MOV)

Video S10 Env lacking the cytoplasmic tail fails to direct assembly towards sites of cell-cell contact. Time-lapse analysis as above was performed for cells expressing mutant Env lacking the cytoplasmic tail. The statistical data for this video can be found in Table S3. The video is played at 10 frames/s and corresponds to Figure 11F.
(6.46 MB MOV)

Video S11 Polarized assembly in the context of the dynamics of cell-to-cell transmission. A COS-1 cell generating MLV labeled with Gag-YFP (red) and expressing dynamin2-CFP (green) in contact with XC target cell expressing mCAT1-CFP (green) was imaged over a period of 8 h and 28 min. A *z*-stack of red and green images was taken every 134 s and merged into a single file to generate Video S11. Repeated rounds of establishment of contact, contact-induced virus assembly, and virus transmission are observed. The video is played at 5 frames/s. Video S11 represents the original data source for the analysis presented in Figure 12.
(20.09 MB MOV)

Video S12 Split channels for Video S11. This time-lapse video depicts red and green channels of Video S11 in parallel.
(17.52 MB MOV)

## Abbreviations

HTLV-1: human T cell leukemia virus 1
MLV: murine leukemia virus
