# Supplementary figures and images for: Assembly of the Murine Leukemia Virus Is Directed towards Sites of Cell–Cell Contact
Source: PLoS Biol. 2009 Jul 28;7(7):e1000163. doi: 10.1371/journal.pbio.1000163 (PMC2709449; doi:10.1371/journal.pbio.1000163)

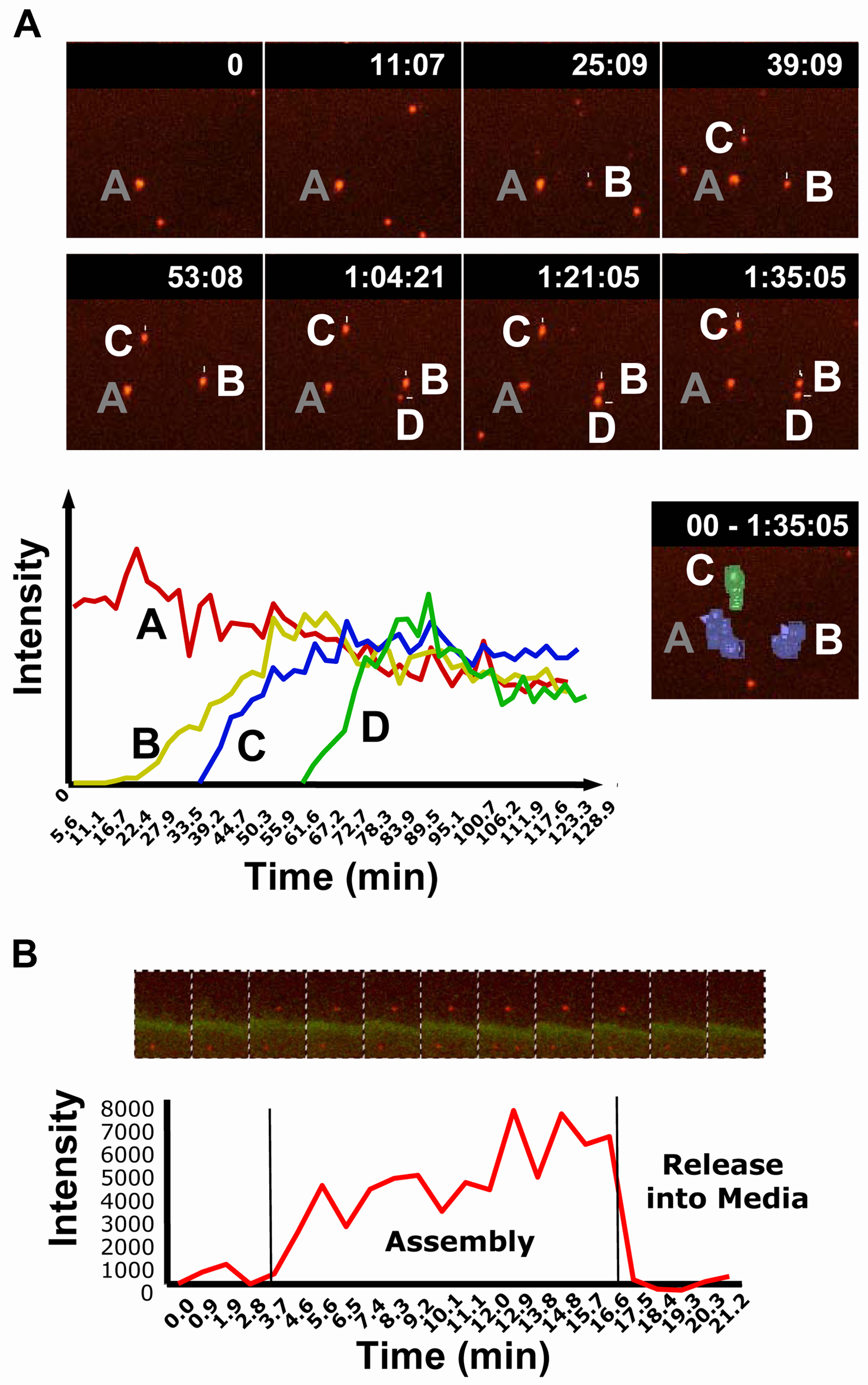

Supplement: Figure S1 — Visualizing MLV assembly and release in Cos-1 cells. (A) An experiment as in (Figure 2A and 2B) was performed in Cos-1 cells generating fluorescently labeled MLV (Gag-YFP, red). (B) Release of a particle from Cos-1 cells into the medium following completion of assembly. (1.70 MB TIF) [file pbio.1000163.s001.tif]

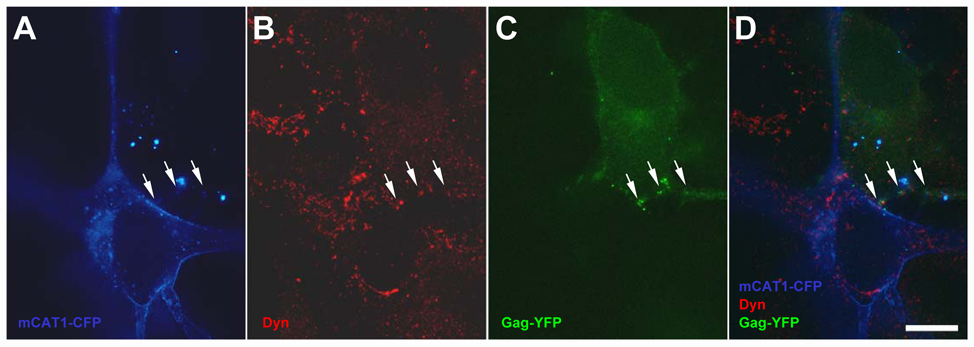

Supplement: Figure S2 — Endogenous dynamin is recruited to sites where target cell membranes are anchored in infected cells. HEK293 cells expressing MLV genome, Env, GagPol, and Gag-YFP (green) were cocultured with XC target cells expressing mCAT1-CFP (blue). Cells were fixed and permeabilized at 3 h post coculture. Endogenous dynamin was stained with dynamin antibody and Alexa568 conjugated secondary antibody (red). Endogenous dynamin localized to sites of cell-cell contact where receptor and virus particles accumulated. Size bars correspond to 15 µm. (0.42 MB TIF) [file pbio.1000163.s002.tif]

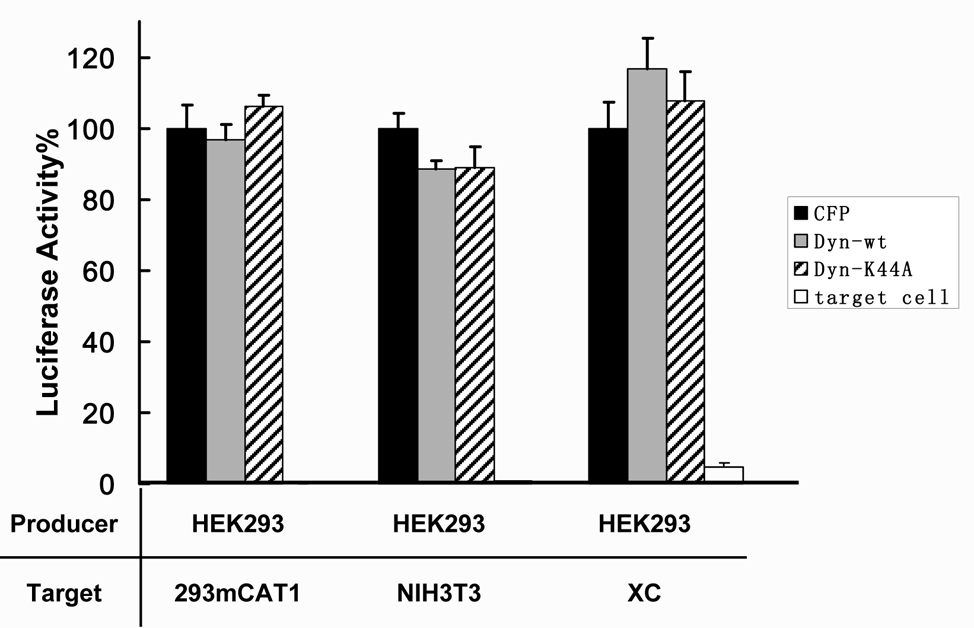

Supplement: Figure S3 — Expression of wild-type and dominant-negative dynamin2 does not affect MLV cell-to-cell transmission. A control experiment as in Figure 3 was performed to determine potential effects of dynamin2 expression on the efficiency of virus cell-to-cell transmission. Producer cells (HEK293 cells) expressing an intron-regulated MLV luciferase reporter inLuc, MLV GagPol, and MLV Env, as well as either wild-type dynamin2-CFP or K44A dynamin2-CFP or CFP control, were cocultured with target cells (XC, NIH 3T3, HEK293 cells stably expressing mCAT1). The luciferase activity originating from infection of target cells is presented. (0.11 MB TIF) [file pbio.1000163.s003.tif]
