# Supplementary material for: Assembly of the Murine Leukemia Virus Is Directed towards Sites of Cell–Cell Contact
Source: PLoS Biol. 2009 Jul 28;7(7):e1000163. doi: 10.1371/journal.pbio.1000163 (PMC2709449; doi:10.1371/journal.pbio.1000163)
Supplement: Table S1 — Calculation of the fold enhancement of MLV assembly at sites of cell-cell contact as presented in Figure 6. Single-particle tracking was applied to identify all de novo assembly events in the MLV-producing HEK293 cell cocultured with XC-expressing mCAT1-CFP in Video S3. Number of assembly events as well as the surface area of contact and the noncontact zones are listed for each frame when initiation of de novo assembly can be detected. The surface area of contact and the noncontact zones in the merged image of all the frames were also listed. To determine the assembly frequency in the absence or presence of cell contact, the number of assembly events observed inside or outside of contact zones was normalized to the respective surface area. For frame-by-frame analysis, average surface area was used, and for overlay analysis, surface area in the merged image was used. To obtain the fold enhancement of MLV assembly in zones of cell-cell contact, the assembly frequency in the presence of cell-cell contact was divided by the assembly frequency in the absence of contact. (0.17 MB PDF) [file pbio.1000163.s004.pdf]

Table S1. Calculation of Enhancement of MLV Assembly at Cell-Cell Contact Sites in Movie 3.

	Contact		Non-contact	
Frame	Assembly Events#	Surface Area(μm^2)	Assembly Events#	Surface Area(μm^2)
1	1	112.63	1	1029.919892
3	0	147.4897	1	982.789455
6	1	160.0843	1	971.832762
9	2	171.1891	0	1040.349109
10	1	139.7071	0	983.1040898
11	1	124.7527	1	1010.421791
14	1	107.42	0	985.7969932
16	1	110.1869	0	996.068893
17	2	134.4323	0	993.1076246
19	2	115.5172	0	979.6431074
21	2	106.1337	0	1003.832969
22	1	95.8433	0	997.5587811
23	1	120.5606	0	995.9485914
25	1	101.1736	0	1003.314747
26	0	109.6965	1	949.3641381
28	1	104.8937	0	1000.464526
29	1	105.4582	0	1010.847473
30	1	98.26784	0	1016.686724
32	2	87.60727	0	1017.343756
35	1	113.9626	0	989.6188803
36	1	104.2737	0	938.8423813
38	1	84.2851	0	1013.383059
40	4	82.62864	0	974.4979035
41	0	71.28328	2	960.3208311
42	2	71.2185	0	945.6903145
43	1	75.94728	0	971.7772382
46	1	61.83499	0	966.71532
53	1	69.40473	0	971.7957461
57	2	87.4222	0	937.6301121
58	1	76.12311	0	944.8574577
59	1	83.28567	0	952.2051049
61	0	70.82984	1	942.0812686
62	1	92.49337	0	984.6402478
69	2	71.11671	0	935.7978273
73	1	68.50709	0	922.9995954
74	1	58.15191	0	985.3157871
76	1	50.5359	0	876.3226024

	Total Assembly Events#	Average Surface Area(μm^2)	Total Assembly Events#	Average Surface Area(μm^2)
37 Event Frames	**44**	**98.54996**	**8**	**977.9158675**
Events/μm^2	**0.446474**		**0.008181**	
Fold of Enhancement*	54. 57676			
**Overlay Frames**	**Total Assembly Events#**	**Surface Area(μm^2)**	**Total Assembly Events#**	**Surface Area(μm^2)**
1-157	**44**	**294.0262**	**8**	**784.3289491**
Events/μm^2	**0.149647**		**0.010199802**	
Fold of Enhancement*	14. 67151355			

de novo assembled particle numbers; * Ratio of *de novo* assembled particle numbers per unit surface area in contacting region to the one in non-contacting region.
