# Supplementary material for: Assembly of the Murine Leukemia Virus Is Directed towards Sites of Cell–Cell Contact
Source: PLoS Biol. 2009 Jul 28;7(7):e1000163. doi: 10.1371/journal.pbio.1000163 (PMC2709449; doi:10.1371/journal.pbio.1000163)
Supplement: Table S2 — Calculation of the fold enhancement of MLV assembly at sites of cell-cell contact as presented in Figure 7. Single-particle tracking was applied to identify all de novo assembly events in MLV-producing cells (HEK293, COS-1 cells) cocultured with receptor-expressing target cells. To determine the assembly frequency in the absence or presence of cell contact, the number of assembly events observed inside or outside of contact zones was normalized to the respective surface area in the frame merged images. To obtain the fold enhancement of MLV assembly in zones of cell-cell contact, the assembly frequency in the presence of cell-cell contact was divided by the assembly frequency in the absence of contact. In addition, the table lists technical parameters of the underlying time-lapse videos such as the total imaging time and the frame time. (0.16 MB PDF) [file pbio.1000163.s005.pdf]

Table S2. Calculation of Enhancement of MLV Assembly at Cell-Cell Contact Sites.

HEK293-XCmCAT1-CFP	A		B		C	
**Time Lapse Video**	**Contact**	**Non-contact**	**Contact**	**Non-contact**	**Contact**	**Non-contact**
**Total Imaging Time (min)**	150		150		171	
**Frame Time (sec)**	60		60		99	
**Surface Area(μm^2)**	294.0262	784.3289	272.1221	476.5329	239.7239	1317.357
**Assembly Events[#]**	44	8	51	9	21	10
**Events/μm^2**	0.149647	0.0102	0.187416	0.018886	0.087601	0.007591
**Fold of Enhancement***	**14.67**		**9.92**		**11.54**	
**HEK293-XCmCAT1-CFP**	**D**		**E**		**F**	
**Time Lapse Video**	**Contact**	**Non-contact**	**Contact**	**Non-contact**	**Contact**	**Non-contact**
**Total Imaging Time (min)**	108		127		120	
**Frame Time (sec)**	52		56		68	
**Surface Area(μm^2)**	310.8221	1383.736	188.1979	735.4403	520.3689	1022.221
**Assembly Events[#]**	16	5	18	9	51	16
**Events/μm^2**	0.051476	0.003613	0.095644	0.012238	0.098007	0.015652
**Fold of Enhancement***	**14.25**		**7.82**		**6.26**	
**HEK293-XCmCAT1-CFP**	**G**		**H**		**I**	
**Time Lapse Video**	**Contact**	**Non-contact**	**Contact**	**Non-contact**	**Contact**	**Non-contact**
**Total Imaging Time (min)**	141		143		126	
**Frame Time (sec)**	65		47		54	
**Surface Area(μm^2)**	212.1286	1622.905	149.3312	644.2702	145.5834	820.6415
**Assembly Events[#]**	16	14	53	33	38	12
**Events/μm^2**	0.075426	0.008627	0.354916	0.051221	0.261019	0.014623
**Fold of Enhancement***	**8.74**		**6.93**		**17.85**	
**HEK293-XClifeAct-CFP**	**J**		**HEK293-HEK293mCAT1-CFP**		**K**	
**Time Lapse Video**	**Contact**	**Non-contact**	**Time Lapse Video**		**Contact**	**Non-contact**
**Total Imaging Time (min)**	83		**Total Imaging Time (min)**		141	
**Frame Time (sec)**	95		**Frame Time (sec)**		54	
**Surface Area(μm^2)**	322.5626	985.5138	**Surface Area(μm^2)**		261.4893	1240.077
**Assembly Events[#]**	42	18	**Assembly Events[#]**		36	26
**Events/μm^2**	0.130207	0.018265	**Events/μm^2**		0.137673	0.020966
**Fold of Enhancement***	**7.1**		**Fold of Enhancement***		**6.6**	
**Cos-XCmCAT1-CFP**	**L**		**M**		**N**	
**Time Lapse Video**	**Contact**	**Non-contact**	**Contact**	**Non-contact**	**Contact**	**Non-contact**
**Total Imaging Time (min)**	110		203		332	
**Frame Time (sec)**	44		82		132	
**Surface Area(μm^2)**	686.0865	3821.105	557.5471	2403.056	559.37	3052.409
**Assembly Events[#]**	18	5	14	7	12	1
**Events/μm^2**	0.026236	0.001309	0.02511	0.002913	0.021453	0.000328
**Fold of Enhancement***	**20.05**		**8.62**		**65.48**	

[#] *de novo* assembled particle numbers; * Ratio of *de novo* assembled particle numbers per unit surface area in contacting region to the one in non-contacting region.
