# Supplementary material for: Assembly of the Murine Leukemia Virus Is Directed towards Sites of Cell–Cell Contact
Source: PLoS Biol. 2009 Jul 28;7(7):e1000163. doi: 10.1371/journal.pbio.1000163 (PMC2709449; doi:10.1371/journal.pbio.1000163)
Supplement: Table S3 — Calculation of the fold enhancement of MLV assembly observed for cells expressing wild-type or mutant Env lacking the cytoplasmic tail as presented in Figure 11. A calculation as in Table S2 was performed for cells expressing Env carrying a histidine 8 deletion (Env ΔH8) and Env lacking a cytoplasmic tail in addition to the histidine 8 mutation (Env ΔH8ΔCT). (0.17 MB PDF) [file pbio.1000163.s006.pdf]

Table S3. Calculation of Enhancement of MLV Assembly at Cell-Cell Contact Sites for Wild Type Env and Mutant Env Lacking the Cytoplasmic Tail.

HEK293-XC	Env ΔH8							
Time Lapse Video	a		b		c		d	
	Contact	Non-contact	Contact	Non-contact	Contact	Non-contact	Contact	Non-contact
Total Imaging Time (min)	105		114		114		105	
Frame Time (sec)	64		66		66		64	
S(μm^2)	94.50148	778.7766	203.0538	556.3281	353.3256	1201.007	44.44679	937.0749
Assembly Events#	15	16	53	5	38	3	7	8
Events/μm^2	0.158728	0.020545	0.261015	0.008988	0.10755	0.002498	0.157492	0.008537
Fold of Enhancement*	**7.73**		**29.04**		**43.06**		**18.45**	

HEK293-XC	Env ΔH8ΔCT							
Time Lapse Video	e		f		g		h	
	Contact	Non-contact	Contact	Non-contact	Contact	Non-contact	Contact	Non-contact
Total Imaging Time (min)	71		74		92		114	
Frame Time (sec)	77		53		65		65	
S(μm^2)	252.3834	959.451	122.6243	771.3919	15.2968	767.1999	126.9829	622.2828
Particle Number#	3	54	5	26	1	43	1	13
Particle/μm^2	0.011887	0.056282	0.040775	0.033705	0.065373	0.056048	0.007875	0.020891
Fold of Enhancement*	**0.21**		**1.21**		**1.17**		**0.38**	

de novo assembled particle numbers; * Ratio of *de novo* assembled particle numbers per unit surface in contacting region to the one in non-contacting region.
